# [1,2-Bis(pyridin-2-ylmeth­oxy)benzene-κ^4^ *N*,*O*,*O*′,*N*′]bis­(nitrato-κ*O*)cobalt(II)

**DOI:** 10.1107/S1600536811013328

**Published:** 2011-04-16

**Authors:** Nan-Nan Huang, Ying-Hui Yu, Ying Liu, Guang-Feng Hou, Jin-Sheng Gao

**Affiliations:** a Pharmaceutical College, Heilongjiang University of Traditional Chinese Medicine, Harbin 150040, People’s Republic of China; b College of Chemistry and Materials Science, Heilongjiang University, Harbin 150080, People’s Republic of China; c Engineering Research Center of Pesticides of Heilongjiang Province, Heilongjiang University, Harbin 150080, People’s Republic of China

## Abstract

In the title compound, [Co(NO_3_)_2_(C_18_H_16_N_2_O_2_)], the Co^II^ ion is six-coordinated in a distorted octa­hedral environment defined by two O and two N atoms from the ligand and by two O atoms from two nitrate anions. A two-dimensional network parallel to the *ab* plane is built up by C—H⋯O hydrogen bonds, which link adjacent mol­ecules in the crystal structure.

## Related literature

For the synthesis and general backround to flexible pyridyl-based ligands, see: Liu *et al.* (2010*a* ▶,*b* ▶). For a related structure, see: Yu *et al.* (2010 ▶).
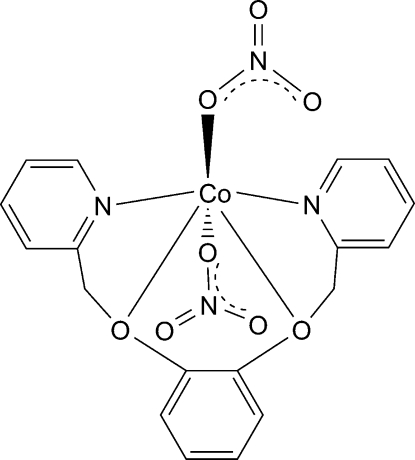


## Experimental

### 

#### Crystal data

[Co(NO_3_)_2_(C_18_H_16_N_2_O_2_)]
*M* *_r_* = 475.28
Triclinic, 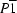

*a* = 8.6281 (17) Å
*b* = 10.701 (2) Å
*c* = 10.921 (2) Å
α = 78.77 (3)°
β = 79.04 (3)°
γ = 78.55 (3)°
*V* = 957.2 (3) Å^3^
*Z* = 2
Mo *K*α radiation
μ = 0.95 mm^−1^
*T* = 291 K
0.24 × 0.21 × 0.19 mm

#### Data collection

Rigaku R-AXIS RAPID diffractometer
Absorption correction: multi-scan (*ABSCOR*; Higashi, 1995 ▶) *T*_min_ = 0.803, *T*_max_ = 0.840
9403 measured reflections
4317 independent reflections
2942 reflections with *I* > 2σ(*I*)
*R*_int_ = 0.033

#### Refinement

*R*[*F* ^2^ > 2σ(*F* ^2^)] = 0.044
*wR*(*F* ^2^) = 0.098
*S* = 1.04
4317 reflections
280 parameters
H-atom parameters constrained
Δρ_max_ = 0.30 e Å^−3^
Δρ_min_ = −0.44 e Å^−3^

### 

Data collection: *RAPID-AUTO* (Rigaku, 1998 ▶); cell refinement: *RAPID-AUTO*; data reduction: *CrystalStructure* (Rigaku/MSC, 2002 ▶); program(s) used to solve structure: *SHELXS97* (Sheldrick, 2008 ▶); program(s) used to refine structure: *SHELXL97* (Sheldrick, 2008 ▶); molecular graphics: *SHELXTL* (Sheldrick, 2008 ▶); software used to prepare material for publication: *SHELXL97*.

## Supplementary Material

Crystal structure: contains datablocks I, global. DOI: 10.1107/S1600536811013328/ng5146sup1.cif

Structure factors: contains datablocks I. DOI: 10.1107/S1600536811013328/ng5146Isup2.hkl

Additional supplementary materials:  crystallographic information; 3D view; checkCIF report

## Figures and Tables

**Table 1:** Hydrogen-bond geometry (Å, °)

*D*—H⋯*A*	*D*—H	H⋯*A*	*D*⋯*A*	*D*—H⋯*A*
C6—H6*A*⋯O3^i^	0.97	2.46	3.241 (3)	138
C13—H13*A*⋯O6^ii^	0.97	2.42	3.296 (4)	150
C17—H17⋯O7^iii^	0.93	2.58	3.469 (4)	160
C18—H18⋯O7	0.93	2.56	2.970 (4)	107

Symmetry codes: (i) 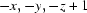; (ii) 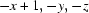; (iii) 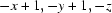.

**Fig. 1.**
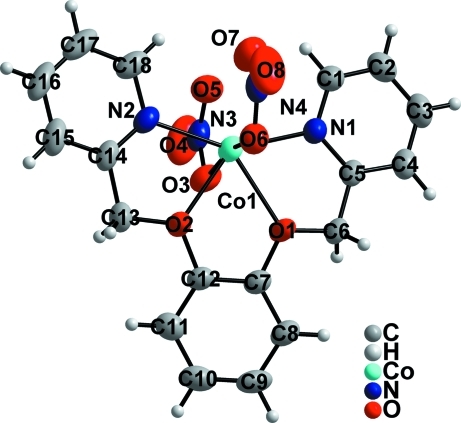
The molecular structure of title compound, showing the atom-labellingscheme and displacement ellipsoids drawn at 50% probability level.

**Fig. 2.**
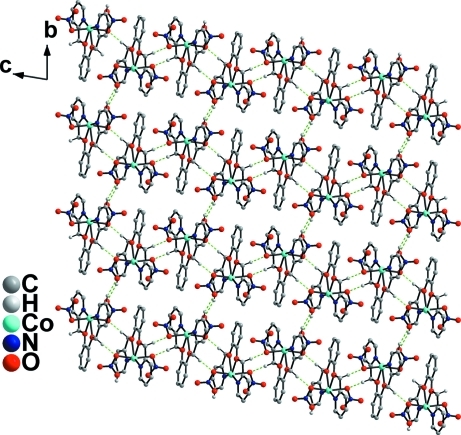
A partial packing view, showing the planar structure parallelled ab plane. Dashed lines indicate the hydrogen bonds and, no involving H atoms have been omitted for clarity.

## supplementary crystallographic information

### Comment

In recent, our group has employed the flexible N-heterocyclic ligands reacting with transition metal to construct several supramolecular architectures (Liu *et al.* 2010*a*, 2010*b*; Yu *et al.* 2010). As a part of our continuing work for bipyridyl aromatic ligands, we report the crystal structure of the title compound here.

1,2-Bis(pyridin-2-ylmethoxy)benzene molecule act as a chelating ligand to coordinate with Co^II^ ion forming a discrete structure. Two nitrate anions also coordinate to the center Co^II^ ion, resulting the Co^II^ ion is six-coordinated in a distorted octahedral environment (Figure 1).

A two-dimensional network, which parallel to *ab* plane, is built up by the C—H···O hydrogen bonds linking these isolated complexes (Figure 2, Tbale 1).

### Experimental

The 1,2-Bis(pyridin-2-ylmethoxy)benzene was synthesized by the reaction of ο-dihydroxybenzene and 2-chloromethylpyridine hydrochloride under nitrogen atmosphere and alkaline condition (Liu *et al.*, 2010*a*). Title ligand (0.58 g, 2 mmol) and Co(NO_3_)_2_^.^H_2_O (0.44 g, 2 mmol) were dissolved in 15 ml e thanol, and then the mixture keep stirring for 30 minute. The resulting solution was filtered, and the filtrate was allowed to stand in a desiccator at room temperature for several days. Red block crystals were obtained.

### Refinement

The reflection data (4 0 5) had been omitted in the refinement. H atoms bound to C atoms were placed in calculated positions and treated as riding on their parent atoms, with C—H = 0.93 Å (aromatic C), C—H = 0.97 Å (methene C), and with *U*_iso_(H) = 1.2*U*_eq_(C).

### Crystal data

**Table:**

[Co(NO_3_)_2_(C_18_H_16_N_2_O_2_)]	*Z* = 2
*M**_r_* = 475.28	*F*(000) = 486
Triclinic, *P*1	*D*_x_ = 1.649 Mg m^−^^3^
Hall symbol: -P 1	Mo *K*α radiation, λ = 0.71073 Å
*a* = 8.6281 (17) Å	Cell parameters from 6221 reflections
*b* = 10.701 (2) Å	θ = 3.0–27.5°
*c* = 10.921 (2) Å	µ = 0.95 mm^−^^1^
α = 78.77 (3)°	*T* = 291 K
β = 79.04 (3)°	Block, red
γ = 78.55 (3)°	0.24 × 0.21 × 0.19 mm
*V* = 957.2 (3) Å^3^	

### Data collection

**Table:**

Rigaku R-AXIS RAPID diffractometer	4317 independent reflections
Radiation source: fine-focus sealed tube	2942 reflections with *I* > 2σ(*I*)
graphite	*R*_int_ = 0.033
ω scans	θ_max_ = 27.5°, θ_min_ = 3.0°
Absorption correction: multi-scan (*ABSCOR*; Higashi, 1995)	*h* = −11→11
*T*_min_ = 0.803, *T*_max_ = 0.840	*k* = −13→13
9403 measured reflections	*l* = −14→12

### Refinement

**Table:**

Refinement on *F*^2^	Primary atom site location: structure-invariant direct methods
Least-squares matrix: full	Secondary atom site location: difference Fourier map
*R*[*F*^2^ > 2σ(*F*^2^)] = 0.044	Hydrogen site location: inferred from neighbouring sites
*wR*(*F*^2^) = 0.098	H-atom parameters constrained
*S* = 1.04	*w* = 1/[σ^2^(*F*_o_^2^) + (0.0345*P*)^2^ + 0.4119*P*] where *P* = (*F*_o_^2^ + 2*F*_c_^2^)/3
4317 reflections	(Δ/σ)_max_ < 0.001
280 parameters	Δρ_max_ = 0.30 e Å^−^^3^
0 restraints	Δρ_min_ = −0.44 e Å^−^^3^

### Special details

**Table:**

Geometry. All e.s.d.'s (except the e.s.d. in the dihedral angle between two l.s. planes) are estimated using the full covariance matrix. The cell e.s.d.'s are taken into account individually in the estimation of e.s.d.'s in distances, angles and torsion angles; correlations between e.s.d.'s in cell parameters are only used when they are defined by crystal symmetry. An approximate (isotropic) treatment of cell e.s.d.'s is used for estimating e.s.d.'s involving l.s. planes.
Refinement. Refinement of *F*^2^ against ALL reflections. The weighted *R*-factor *wR* and goodness of fit *S* are based on *F*^2^, conventional *R*-factors *R* are based on *F*, with *F* set to zero for negative *F*^2^. The threshold expression of *F*^2^ > σ(*F*^2^) is used only for calculating *R*-factors(gt) *etc*. and is not relevant to the choice of reflections for refinement. *R*-factors based on *F*^2^ are statistically about twice as large as those based on *F*, and *R*- factors based on ALL data will be even larger.

### Fractional atomic coordinates and isotropic or equivalent isotropic displacement parameters (Å^2^)

**Table:**

	*x*	*y*	*z*	*U*_iso_*/*U*_eq_	
C1	−0.0687 (3)	0.3713 (3)	0.2950 (3)	0.0486 (7)	
H1	0.0015	0.4286	0.2579	0.058*	
C2	−0.2202 (3)	0.4199 (3)	0.3487 (3)	0.0554 (7)	
H2	−0.2512	0.5083	0.3484	0.066*	
C3	−0.3259 (3)	0.3361 (3)	0.4030 (3)	0.0555 (8)	
H3	−0.4289	0.3667	0.4408	0.067*	
C4	−0.2763 (3)	0.2067 (3)	0.4004 (2)	0.0477 (7)	
H4	−0.3459	0.1484	0.4356	0.057*	
C5	−0.1215 (3)	0.1633 (2)	0.3447 (2)	0.0372 (5)	
C6	−0.0689 (3)	0.0228 (2)	0.3402 (3)	0.0443 (6)	
H6A	−0.0768	−0.0254	0.4255	0.053*	
H6B	−0.1383	−0.0067	0.2956	0.053*	
C7	0.1666 (3)	−0.1270 (2)	0.2818 (3)	0.0440 (6)	
C8	0.0924 (3)	−0.2336 (3)	0.3200 (3)	0.0506 (7)	
H8	−0.0179	−0.2243	0.3452	0.061*	
C9	0.1852 (4)	−0.3553 (3)	0.3201 (3)	0.0591 (8)	
H9	0.1365	−0.4283	0.3450	0.071*	
C10	0.3477 (4)	−0.3692 (3)	0.2842 (3)	0.0573 (8)	
H10	0.4086	−0.4515	0.2868	0.069*	
C11	0.4228 (3)	−0.2613 (3)	0.2436 (3)	0.0504 (7)	
H11	0.5330	−0.2706	0.2177	0.060*	
C12	0.3307 (3)	−0.1406 (2)	0.2426 (2)	0.0425 (6)	
C13	0.5543 (3)	−0.0297 (3)	0.1678 (3)	0.0501 (7)	
H13A	0.5946	−0.0789	0.0992	0.060*	
H13B	0.6077	−0.0716	0.2392	0.060*	
C14	0.5874 (3)	0.1051 (3)	0.1271 (2)	0.0431 (6)	
C15	0.7396 (3)	0.1244 (3)	0.0648 (3)	0.0591 (8)	
H15	0.8175	0.0546	0.0449	0.071*	
C16	0.7728 (4)	0.2473 (4)	0.0331 (3)	0.0686 (10)	
H16	0.8734	0.2624	−0.0089	0.082*	
C17	0.6556 (4)	0.3484 (3)	0.0643 (3)	0.0654 (9)	
H17	0.6768	0.4325	0.0463	0.078*	
C18	0.5062 (4)	0.3233 (3)	0.1226 (3)	0.0542 (7)	
H18	0.4265	0.3924	0.1414	0.065*	
Co1	0.22927 (4)	0.16895 (4)	0.22727 (4)	0.04752 (14)	
N1	−0.0169 (2)	0.2441 (2)	0.29360 (19)	0.0399 (5)	
N2	0.4706 (2)	0.2029 (2)	0.15346 (19)	0.0417 (5)	
N3	0.2919 (2)	0.2222 (3)	0.4483 (2)	0.0494 (6)	
N4	0.1734 (3)	0.2913 (3)	−0.0118 (3)	0.0585 (7)	
O1	0.0897 (2)	0.00028 (17)	0.2786 (2)	0.0574 (6)	
O2	0.3876 (2)	−0.02515 (17)	0.2020 (2)	0.0546 (5)	
O3	0.2818 (2)	0.1180 (2)	0.41373 (19)	0.0591 (5)	
O4	0.3220 (3)	0.2203 (3)	0.5536 (2)	0.0902 (9)	
O5	0.2706 (3)	0.3219 (2)	0.3710 (2)	0.0711 (6)	
O6	0.1839 (2)	0.1745 (2)	0.04378 (19)	0.0600 (5)	
O7	0.1811 (3)	0.3725 (3)	0.0520 (3)	0.0840 (8)	
O8	0.1569 (3)	0.3154 (3)	−0.1235 (2)	0.1009 (10)	

### Atomic displacement parameters (Å^2^)

**Table:**

	*U*^11^	*U*^22^	*U*^33^	*U*^12^	*U*^13^	*U*^23^
C1	0.0457 (15)	0.0407 (15)	0.0580 (17)	−0.0091 (12)	−0.0044 (13)	−0.0063 (13)
C2	0.0514 (16)	0.0496 (17)	0.0618 (18)	0.0043 (13)	−0.0104 (14)	−0.0126 (14)
C3	0.0373 (14)	0.070 (2)	0.0531 (17)	0.0016 (14)	−0.0013 (13)	−0.0109 (15)
C4	0.0342 (13)	0.0594 (18)	0.0448 (15)	−0.0084 (12)	−0.0036 (11)	0.0005 (13)
C5	0.0324 (12)	0.0445 (14)	0.0340 (12)	−0.0096 (10)	−0.0045 (10)	−0.0021 (11)
C6	0.0319 (12)	0.0455 (15)	0.0522 (15)	−0.0147 (11)	0.0007 (11)	0.0009 (12)
C7	0.0453 (14)	0.0359 (14)	0.0508 (15)	−0.0122 (11)	−0.0026 (12)	−0.0061 (12)
C8	0.0515 (15)	0.0435 (16)	0.0602 (17)	−0.0209 (13)	−0.0058 (13)	−0.0064 (13)
C9	0.080 (2)	0.0359 (15)	0.068 (2)	−0.0218 (15)	−0.0189 (17)	−0.0047 (14)
C10	0.074 (2)	0.0354 (15)	0.0637 (19)	−0.0036 (14)	−0.0168 (16)	−0.0099 (13)
C11	0.0520 (16)	0.0444 (16)	0.0535 (16)	−0.0015 (13)	−0.0103 (13)	−0.0094 (13)
C12	0.0447 (14)	0.0338 (13)	0.0496 (15)	−0.0117 (11)	−0.0034 (12)	−0.0070 (11)
C13	0.0313 (12)	0.0565 (17)	0.0586 (17)	−0.0069 (12)	−0.0021 (12)	−0.0053 (14)
C14	0.0326 (12)	0.0611 (17)	0.0369 (13)	−0.0166 (12)	−0.0047 (10)	−0.0032 (12)
C15	0.0362 (14)	0.091 (2)	0.0493 (16)	−0.0195 (15)	−0.0026 (12)	−0.0036 (16)
C16	0.0487 (17)	0.106 (3)	0.0537 (18)	−0.0442 (19)	−0.0098 (14)	0.0134 (19)
C17	0.076 (2)	0.075 (2)	0.0544 (18)	−0.0507 (19)	−0.0187 (16)	0.0144 (16)
C18	0.0658 (18)	0.0554 (18)	0.0467 (15)	−0.0321 (15)	−0.0092 (14)	0.0016 (13)
Co1	0.03625 (19)	0.0440 (2)	0.0571 (2)	−0.01247 (15)	0.00876 (16)	−0.00555 (17)
N1	0.0346 (10)	0.0406 (12)	0.0432 (12)	−0.0088 (9)	−0.0029 (9)	−0.0040 (9)
N2	0.0417 (11)	0.0469 (13)	0.0372 (11)	−0.0196 (10)	−0.0020 (9)	−0.0005 (9)
N3	0.0327 (11)	0.0671 (17)	0.0470 (14)	−0.0159 (11)	0.0004 (10)	−0.0050 (13)
N4	0.0333 (11)	0.0727 (19)	0.0576 (16)	−0.0093 (12)	0.0030 (11)	0.0084 (14)
O1	0.0376 (9)	0.0350 (10)	0.0902 (15)	−0.0131 (8)	0.0185 (10)	−0.0082 (10)
O2	0.0345 (9)	0.0371 (10)	0.0862 (14)	−0.0092 (8)	0.0084 (9)	−0.0098 (9)
O3	0.0589 (12)	0.0505 (12)	0.0645 (13)	−0.0210 (10)	0.0053 (10)	−0.0035 (10)
O4	0.0743 (16)	0.159 (3)	0.0483 (13)	−0.0396 (17)	−0.0144 (12)	−0.0176 (15)
O5	0.0630 (13)	0.0517 (13)	0.0877 (16)	−0.0118 (10)	−0.0046 (12)	0.0102 (12)
O6	0.0488 (11)	0.0600 (13)	0.0646 (13)	−0.0102 (10)	0.0015 (10)	−0.0032 (11)
O7	0.0663 (15)	0.0733 (17)	0.114 (2)	−0.0218 (13)	−0.0009 (14)	−0.0223 (16)
O8	0.0746 (16)	0.156 (3)	0.0518 (14)	−0.0088 (17)	−0.0101 (12)	0.0219 (16)

### Geometric parameters (Å, °)

**Table:**

C1—N1	1.347 (3)	C13—O2	1.410 (3)
C1—C2	1.371 (4)	C13—C14	1.492 (4)
C1—H1	0.9300	C13—H13A	0.9700
C2—C3	1.379 (4)	C13—H13B	0.9700
C2—H2	0.9300	C14—N2	1.335 (3)
C3—C4	1.370 (4)	C14—C15	1.394 (3)
C3—H3	0.9300	C15—C16	1.366 (5)
C4—C5	1.388 (3)	C15—H15	0.9300
C4—H4	0.9300	C16—C17	1.373 (5)
C5—N1	1.343 (3)	C16—H16	0.9300
C5—C6	1.489 (4)	C17—C18	1.378 (4)
C6—O1	1.403 (3)	C17—H17	0.9300
C6—H6A	0.9700	C18—N2	1.348 (3)
C6—H6B	0.9700	C18—H18	0.9300
C7—C8	1.375 (4)	Co1—O6	2.101 (2)
C7—C12	1.386 (4)	Co1—O3	2.114 (2)
C7—O1	1.388 (3)	Co1—N1	2.156 (2)
C8—C9	1.387 (4)	Co1—N2	2.159 (2)
C8—H8	0.9300	Co1—O2	2.2825 (19)
C9—C10	1.369 (4)	Co1—O1	2.2876 (19)
C9—H9	0.9300	N3—O4	1.223 (3)
C10—C11	1.392 (4)	N3—O5	1.230 (3)
C10—H10	0.9300	N3—O3	1.268 (3)
C11—C12	1.374 (4)	N4—O8	1.226 (3)
C11—H11	0.9300	N4—O7	1.233 (4)
C12—O2	1.382 (3)	N4—O6	1.272 (3)
			
N1—C1—C2	123.0 (3)	C16—C15—C14	119.1 (3)
N1—C1—H1	118.5	C16—C15—H15	120.5
C2—C1—H1	118.5	C14—C15—H15	120.5
C1—C2—C3	119.1 (3)	C15—C16—C17	119.1 (3)
C1—C2—H2	120.4	C15—C16—H16	120.5
C3—C2—H2	120.4	C17—C16—H16	120.5
C4—C3—C2	118.7 (3)	C16—C17—C18	119.1 (3)
C4—C3—H3	120.7	C16—C17—H17	120.5
C2—C3—H3	120.7	C18—C17—H17	120.5
C3—C4—C5	119.5 (3)	N2—C18—C17	122.8 (3)
C3—C4—H4	120.2	N2—C18—H18	118.6
C5—C4—H4	120.2	C17—C18—H18	118.6
N1—C5—C4	122.1 (2)	O6—Co1—O3	167.12 (8)
N1—C5—C6	118.6 (2)	O6—Co1—N1	92.66 (8)
C4—C5—C6	119.3 (2)	O3—Co1—N1	91.78 (8)
O1—C6—C5	110.0 (2)	O6—Co1—N2	90.83 (8)
O1—C6—H6A	109.7	O3—Co1—N2	91.54 (8)
C5—C6—H6A	109.7	N1—Co1—N2	149.17 (8)
O1—C6—H6B	109.7	O6—Co1—O2	85.45 (9)
C5—C6—H6B	109.7	O3—Co1—O2	83.28 (9)
H6A—C6—H6B	108.2	N1—Co1—O2	139.03 (8)
C8—C7—C12	120.7 (2)	N2—Co1—O2	71.79 (8)
C8—C7—O1	125.1 (2)	O6—Co1—O1	84.09 (9)
C12—C7—O1	114.2 (2)	O3—Co1—O1	85.79 (8)
C7—C8—C9	118.7 (3)	N1—Co1—O1	72.04 (7)
C7—C8—H8	120.6	N2—Co1—O1	138.79 (8)
C9—C8—H8	120.6	O2—Co1—O1	67.05 (6)
C10—C9—C8	120.7 (3)	C5—N1—C1	117.6 (2)
C10—C9—H9	119.6	C5—N1—Co1	120.29 (17)
C8—C9—H9	119.6	C1—N1—Co1	121.93 (17)
C9—C10—C11	120.6 (3)	C14—N2—C18	117.5 (2)
C9—C10—H10	119.7	C14—N2—Co1	120.61 (17)
C11—C10—H10	119.7	C18—N2—Co1	121.64 (19)
C12—C11—C10	118.6 (3)	O4—N3—O5	123.2 (3)
C12—C11—H11	120.7	O4—N3—O3	120.1 (3)
C10—C11—H11	120.7	O5—N3—O3	116.7 (2)
C11—C12—O2	125.1 (2)	O8—N4—O7	124.7 (3)
C11—C12—C7	120.6 (2)	O8—N4—O6	118.6 (3)
O2—C12—C7	114.3 (2)	O7—N4—O6	116.7 (3)
O2—C13—C14	108.7 (2)	C7—O1—C6	117.91 (19)
O2—C13—H13A	110.0	C7—O1—Co1	121.70 (15)
C14—C13—H13A	110.0	C6—O1—Co1	118.14 (15)
O2—C13—H13B	110.0	C12—O2—C13	118.1 (2)
C14—C13—H13B	110.0	C12—O2—Co1	121.94 (14)
H13A—C13—H13B	108.3	C13—O2—Co1	118.68 (16)
N2—C14—C15	122.4 (3)	N3—O3—Co1	106.43 (17)
N2—C14—C13	118.6 (2)	N4—O6—Co1	107.7 (2)
C15—C14—C13	118.9 (3)		

### Hydrogen-bond geometry (Å, °)

**Table:**

*D*—H···*A*	*D*—H	H···*A*	*D*···*A*	*D*—H···*A*
C6—H6A···O3^i^	0.97	2.46	3.241 (3)	138
C13—H13A···O6^ii^	0.97	2.42	3.296 (4)	150
C17—H17···O7^iii^	0.93	2.58	3.469 (4)	160
C18—H18···O7	0.93	2.56	2.970 (4)	107

Symmetry codes: (i) −*x*, −*y*, −*z*+1; (ii) −*x*+1, −*y*, −*z*; (iii) −*x*+1, −*y*+1, −*z*.
